# Guards at the gate: physiological and pathological roles of tissue-resident innate lymphoid cells in the lung

**DOI:** 10.1007/s13238-017-0379-5

**Published:** 2017-03-07

**Authors:** Hang Cheng, Chengyan Jin, Jing Wu, Shan Zhu, Yong-Jun Liu, Jingtao Chen

**Affiliations:** 1 Institute of Translational Medicine, The First Hospital, Jilin University, Changchun, 130061 China; 2 Department of Pediatrics, The First Hospital, Jilin University, Changchun, 130021 China; 3 Department of Thoracic Surgery, The Second Hospital, Jilin University, Changchun, 130041 China; 4 Sanofi Research and Development, Cambridge, MA 02139 USA

**Keywords:** lung, innate lymphoid cells, pulmonary diseases, regional immunity

## Abstract

The lung is an important open organ and the primary site of respiration. Many life-threatening diseases develop in the lung, e.g., pneumonia, asthma, chronic obstructive pulmonary diseases (COPDs), pulmonary fibrosis, and lung cancer. In the lung, innate immunity serves as the frontline in both anti-irritant response and anti-tumor defense and is also critical for mucosal homeostasis; thus, it plays an important role in containing these pulmonary diseases. Innate lymphoid cells (ILCs), characterized by their strict tissue residence and distinct function in the mucosa, are attracting increased attention in innate immunity. Upon sensing the danger signals from damaged epithelium, ILCs activate, proliferate, and release numerous cytokines with specific local functions; they also participate in mucosal immune-surveillance, immune-regulation, and homeostasis. However, when their functions become uncontrolled, ILCs can enhance pathological states and induce diseases. In this review, we discuss the physiological and pathological functions of ILC subsets 1 to 3 in the lung, and how the pathogenic environment affects the function and plasticity of ILCs.

## Introduction

Regional immunity greatly differs from the conventional immune organ or system. Because human diseases are tightly connected with regional immunity, researchers have recently begun to focus on the regional immunity of organs such as the lung, intestine, liver, and skin. The lung is an open organ that is involved in gas conduction and exchange. Approximately 8,000 to 9,000 liters of air are breathed into the lung every day. Compared with the gut and the skin, the lung has a wider surface area, up to 90 m^2^. A single layer of pulmonary epithelial cells covers the alveoli (Kopf et al., 2015). Because of these characteristics, the lung is constantly exposed to environmental stressors, such as pathogens, allergens, and airborne toxins, e.g., cigarette smoke. In the battle between the mucosal immune cells and the invaders, the innate immune cells are the first line of defense, fortifying the trenches. The innate immune cells in the lung mainly comprise lung-resident macrophages, lung-resident dendritic cells (DCs) (Holt et al., 2008; Kopf et al., 2015), and emerging sets of innate lymphoid cells (ILCs) (Spits and Di Santo, 2011; Spits and Cupedo, 2012; Eberl et al., 2015).

ILCs are important tissue-resident innate immune cells. They are promptly activated by danger signals from injured mucosa and produce an array of effective cytokines to repel pathogens and tumor cells, thereby maintaining mucosal integrity. However, if they are excessively activated, they may cause pathologic tissue damage, e.g., asthma, Crohn’s disease, or psoriasis (Buonocore et al., 2010; Spits and Cupedo, 2012; Spits et al., 2013; Karta et al., 2016). Research into ILCs in the lung is only now in its infancy but it is known that there are three groups of ILCs in the lung, namely ILC1s, ILC2s, and ILC3s. Recently Lai et al. reviewed the origin, development, heterogeneity, and interaction of ILCs with other cells in the lung (Lai et al., 2016), however the roles of ILCs in lung pathologies have not been extensively reviewed, especially with respect to ILC1s and ILC3s. With this in mind, here we describe the general characteristics, functions, and phenotypic plasticity of ILCs, focusing especially on the roles of all three groups of ILCs in diseases of the lung.

## CLASSIFICATION AND GENERAL CHARACTERISTICS OF ILCs

Lymphoid tissue inducer (LTi) cells and NK cells are prototypic ILCs, which require the common γ chain of the interleukin-2 receptor (IL-2Rγ) and transcriptional repressor inhibitor of DNA binding 2 (Id2) for their development (Kelly and Scollay, 1992; Held et al., 2011; Hesslein and Lanier, 2011). Over the last ten years, numerous cells have been identified whose development is also IL-2Rγ and Id2-dependent; these cells are referred to as “innate lymphoid cells” (ILCs). ILCs have three main characteristics: a lymphoid morphology, the absence of rearranged antigen-specific receptors, and a lack of myeloid dendritic cell phenotypical markers; ILCs also do not express antigen receptors or undergo clonal selection (Spits and Cupedo, 2012; Spits et al., 2013). In 2013, Spits et al. classified the ILCs into three groups according to their cytokine secretion ability, mirroring CD4^+^ T helper (Th) cells: group 1 ILCs (ILC1s), comprising conventional NK cells and ILCs that produce interferon-γ (IFN-γ); group 2 ILCs (ILC2s), which are ILCs that secrete type 2 cytokines, such as IL-5 and IL-13; and group 3 ILCs (ILC3s), that produce IL-17 and/or IL-22 (Spits and Cupedo, 2012; Spits et al., 2013).

ILCs mirror Th cells but also differ from them. Although ILCs and Th cells both arise from a common lymphoid progenitor (CLP), the development of ILCs is unique. It is generally believed that ILCs initially develop in fetal liver, whereas after birth they develop in the bone marrow (Vosshenrich et al., 2005; Sawa et al., 2010; Klose et al., 2014), and are then subsequently recruited into other tissues (Eberl et al., 2015; Gasteiger et al., 2015). Migration into tissues is likely mediated by the co-ordinated action of adhesion molecules and chemokines (Eberl et al., 2015). Interestingly, some researchers found that ILC progenitors seed themselves in tissues in the embryonic and adult phases, and in these tissue micro-environments they undergo development and differentiation (Montaldo et al., 2014; Bando et al., 2015). It should be noted that, in contrast to Th cells, ILCs remain tissue-resident; they are maintained locally in one organ and do not re-enter the circulation or migrate to other organs (Gasteiger et al., 2015; Fan and Rudensky, 2016). Following acute environmental challenges tissue-resident ILCs expand locally and in this way the pool of cells is renewed, although phenotypic transformation can also occur. Hematogenously derived ILC precursors or mature ILCs can also partially supply the local tissue ILC pool (Gasteiger et al., 2015). In addition, ILCs lack recombination-activating gene (RAG), meaning that unlike B and T cells, ILCs can be activated directly (Spits and Cupedo, 2012; Spits et al., 2013). When the mucosa is invaded by pathogens, allergens, or tumor cells, damaged epithelial cells secrete cytokines to directly stimulate ILCs. ILCs then become promptly activated, proliferate, and produce copious amounts of cytokines to repel the invaders and maintain mucosal homeostasis; this response, however, may also lead to pathological damage (Spits and Di Santo, 2011; Scanlon and McKenzie, 2012; Philip and Artis, 2013; Salimi and Ogg, 2014). Thus, ILCs become activated by sensing danger signals from the tissue milieu rather than by antigen presentation with antigen-presenting cells (APCs) (Drake and Kita, 2014; Eberl et al., 2015). Compared with the few days or weeks required by Th cells (Hansen et al., 1999), ILCs can therefore be activated more quickly (Spits and Cupedo, 2012; Fan and Rudensky, 2016) (Table 1).

**Table 1:** Comparison of ILC and Th cell characteristics

	ILCs	TH cells
TF	ILC1s: T-bet ILC2s: Gata3 ILC3s: RORγt	Th1: T-bet Th2: Gata3 Th17: RORγt
Principal effector cytokines	ILC1s: IFN-γ ILC2s: IL-3, IL-4, IL-9, IL-13 ILC3s: IL-17, IL-22	Th1: IFN-γ Th2: IL-3, IL-4, IL-9, IL-13 Th17: IL-17, IL-22
Genesis	CLP	CLP
Innate/Adaptive system	Innate immune cells	Adaptive immune cells
RAG	Absent	Present
Response time	Hours~days	Days~weeks
Activated pathway	APC-independent Directly activated	APC-dependent Indirectly activated
Tissue residency	Yes	No
Extension/Activation	Tissue	Lymph node
Recruitment (Back to circulation)	Seldom	Frequently
Memory-property	Antigen non-specific memory-property	Antigen specific memory-property

APC, antigen-presenting cell; CLP, common lymphoid progenitor; ILCs, innate lymphoid cells; SLO, secondary lymph organ; TF, transcription factor

## ILC plasticity

**Figure 1:**
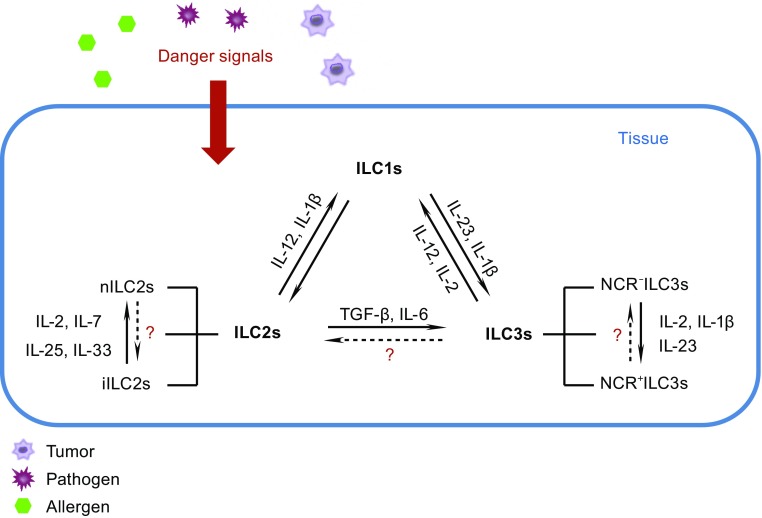
**ILC plasticity**. ILCs recruit into the lung and become resident in the mucous epithelium. When the tissue is exposed to danger signals elicited by pathogens, allergens or tumor cells, the epithelium or other innate immune cells produce many cytokines. In response to these cytokines, ILCs may alter their phenotype to respond to the environment. IL-2 and IL-12 drive the transformation of ILC3s to ILC1s. ILC1s convert to ILC3s under the influence of IL-1β and IL-23; ILC2s also transform to ILC1s when cultured with IL-12 and IL-1β. Upon increased GATA3 expression, ILC1s gain ILC2s characteristics; when cultured with TGF-β and IL-6, ILC2s become ILC3-like. Whether ILC3s convert into ILC2s is still unclear. In the ILC2 and ILC3 sub-groups, iILC2 cells give rise to cells with nILC2 phenotype when cultured in the presence of IL-2, IL-7, IL-25, and IL-33 *in vitro* or *in vivo*. Under the influence of IL-2, IL-1β, and IL-23, NCR^−^ILC3s express NCR^+^. The hypothesis that nILC2s convert to iILC2s and NCR^+^ILC3s convert to NCR^−^ILC3s should be confirmed in the future. See text for details

The phenotype of ILCs is not stable; these cells are highly plastic and can change phenotypes under the influence of the environment. IL-2 and IL-12 drive human natural cytotoxicity receptor (NCR)-positive ILC3s (NCR^+^ILC3s) to transform into ILC1s (Cella et al., 2010; Bernink et al., 2013; Bernink et al., 2015). In response to the tissue environment *in vivo*, ILC3s down-regulate the expression of RORγt (retinoic acid receptor-related orphan receptor γt) and produce IFN-γ (Vonarbourg et al., 2010a). IL-1β and IL-12 induce ILC2s to express T-bet and to produce IFN-γ while down-regulating ST2 and GATA3, and losing the ability to produce IL-5 and IL-13 (Bal et al., 2016; Kim et al., 2016; Lim et al., 2016). IL-23 and IL-1β cause CD127^+^ ILC1s to differentiate into IL-22-producing ILC3s (Bernink et al., 2015). Enhanced GATA3 expression by ILC1s results in their conversion to ILC2s with the capacity to produce greater amounts of type 2 cytokines (Mjosberg et al., 2012; KleinJan et al., 2014). Such plasticity occurs not only between ILC groups, but also between ILC subgroups. For example, mouse NKp46^−^RORγt^+^ LTi-like cells may transform into NKp46^+^RORγt^+^ cells either *in vivo* or *in vitro* (Vonarbourg et al., 2010a; Klose et al., 2013; Rankin et al., 2013; Rankin et al., 2016). Human NKp44^−^ILC3s undergo a profound shift toward NKp44^+^ ILC3s upon culture in the presence of IL-2, IL-1β, and IL-23, and they display pro-inflammatory properties (Bernink et al., 2013; Glatzer et al., 2013). Plasticity is one of the important characteristics of ILCs, and this property is especially important in the lung; the shift of ILC2s to ILC3s and the plasticity within ILC2 subgroups will be discussed below in detail (Table 2) (Fig. 1).

**Table 2:** Characteristics of lung ILCs

	ILC1s	ILC2s	ILC3s
Development-dependent TF	T-bet	Gata3, RORα, EST1, Bcl11b, G9a, Gfi1	RORγt
Surface marker (Human)	CD127^high^ILC1s: Lin^−^ CD127^+^ CD117^−^ NKp44^−^ CD25^−^ CD103^−^ CCR6^−^ CD127^low^ILC1s: Lin^−^ CD127^−^ NKp44^−^ NKp46^+^ CD103^+^	Lin^−^ CD127^+^ CRTH2^+^ CD117^−/+^ CD161^+^ CD25^+^ ICOS^+^ ST2^+^	Lin^−^ CD127^+^ CD117^+^ CD161^+^ CD25^+^ NKp44^−/+^ NKp46^−/+^ NKp30^−/+^ CD56^−/+^ CCR6^−/+^ CXCR5^+^
Surface marker (Mouse)	Lin^−^ CD90^+^ IL-12R2^+^ IL-18Rα^+^	Lin^−^ CD90^+^ CD127^+^ ST2^+^ Sca-1^+^ KLRG1^+^ CD25^+^ IL-7RB^+^ CD44^+^ IL-9R^+^	Lin^−^ CD90^+^ CD127^+^ NKp46^−/+^ NKp30^−/+^ CD56^−/+^ CCR6^−/+^
Active factors	IL-12, IL-18	IL-25, IL-33, TSLP, PGD_2_, TGF-β, Spred1, Arginase 1, TL1A, RAGE, SP-D, IRF4 CysLT1, IL-1β	IL-1β, IL-23
Inhibitory factors	-	IFN-γ, IL-27, Lipoxin4 Corticosteroid, TSA, PGI_2_, HES	-
Effective cytokines	IFN-γ	IL-4, IL-5, IL-6, IL-13, IL-9	IL-17, IL-22, TNF-α, IL-8, IL-2, GM-CSF, lymphotoxin

NCR, natural cytotoxicity receptor; TF, transcription factor; Bcl11b, B cell leukemia/lymphoma 11b; Gfi1, growth factor independence-1; Lin, lineage; G9a, lysine methyltransferase G9a; HES, *Heligmosomoides polygyrus* excretory/secretory products; TSLP, thymic stromal lymphopoietin; PGD_2_, prostaglandin D_2_; TL1A, tumor necrosis factor like cytokine 1A; RAGE, receptor for advanced glycation end-products; SP-D, surfactant protein D; IRF4, interferon regulatory factor 4; TSA, trichostatin A; PGI_2_, prostaglandin I_2_; CysLT1, cysteinyl leukotriene receptor 1

## Identification and characterization of ILC1s

Compared with the other ILC groups, ILC1s are the least studied. Their characteristics are not yet well defined, and indeed there is even still debate concerning their classification. As of now, the ILCs nomenclature introduced by Spits et al. (2013) is widely accepted, with NK cells and ILC1s belonging to group 1 ILCs. Group 1 ILCs are defined by their ability to produce IFN-γ and inability to produce IL-4, IL-13, IL-22, and IL-17. They require transcription factor T-bet for their development (Spits et al., 2013). NK cells have been reviewed elsewhere, and therefore we will only focus on ILC1s in this review.

The original report concerning mouse ILC1s described them as RORγt^−^NKR^−^LTi, capable of releasing IFN-γ, and as being potent inducers of experimental colitis (Vonarbourg et al., 2010b). Another group of mouse ILC1s was then described as NKp46^+^NKp1.1^+^Eomes^−^T-bet^+^, with the ability to produce IFN-γ (Klose et al., 2014). In humans, ILC1s can be divided into CD127^low^ ILC1s and CD127^high^ ILC1s, based on the expression of CD127. CD127^low^ ILC1s are defined as CD3^−^CD56^+^NKp44^+^CD103^+^ and CD3^−^CD56^+^NKp44^−^CD103^−^; the former have an intraepithelial localization (Fuchs et al., 2013). CD127^high^ ILC1s were first identified in human tonsil and intestine, and they are defined as Lineage (Lin)^−^CD127^+^CRTH2^+^CD117^−^NKp44^−^ (Bernink et al., 2013). Although the markers that define ILC1s in mouse and human are somewhat different, the two are generally defined as lineage negative, T-bet positive, and both are capable of producing IFN-γ.

CD127^high^ and CD127^low^ ILC1s have also been identified in the human lung. CD127^high^ ILC1s are identified as Lin^−^CD127^+^CD117^−^NKp44^−^; they express T-bet but do not express C-C motif chemokine receptor 6 (CCR6), CD103, or CD25. CD127^low^ ILC1s are defined as Lin^−^CD127^−^NKp46^+^, and express T-bet. Similar to CD127^high^ ILC1s in the tonsil and the intestine, most Lin^−^CD127^−^NKp46^+^NKp44^+^cells express CD103 (Carrega et al., 2015). Another group identified non-toxic ILC1s in the human lung was Lin^−^CD56^−^IL12Rβ2^+^ (De Grove et al., 2016). In mouse lung, ILC1s are defined by the Lin^−^CD90^+^T-bet^+^ phenotype, and they express IL-12Rβ2 and IL-18Rα; furthermore, IL-12 and IL-18 enhance ILC1 expansion *in vivo*, and they produce copious amounts of IFN-γ (Silver et al., 2016a) (Table 2).

## ILC1s in the lung

### ILC1s and infection

ILC1s produce large amounts of IFN-γ and protect the organism against pathogens as first reported in mouse models of intracellular infection, e.g., infections caused by the parasite *Toxoplasma gondii* and by *Clostridium difficile* in the intestine (Klose et al., 2014; Abt et al., 2015). Silver et al. (2016a, b) found that during lung infection in mice caused by either influenza A, *Haemophilus influenzae*, respiratory syncytial virus (RSV), or *Staphylococcus aureus*, GATA3 expression in the resident ILC2s was rapidly down-regulated (within two days after infection), and this was accompanied by decreased expression of ST2, CD25 (IL-2Rα), IL-7Rα, inducible costimulator (ICOS), and the stem cell factor receptor c-kit (CD117). Meanwhile, the T-bet^+^ ILC1 number in the lung increased and the expression of IL-12 and IL-18 receptors (IL-12Rβ2 and IL-18Rα) in ILC1s was up-regulated. The down-regulation of GATA3 expression was negatively correlated with IL-18Rα up-regulation. These results indicate that during infection of the lung, ILC2s may lose their properties and phenotypically convert into ILC1s. To confirm these findings, Silver et al. used a reporter mouse that expressed ST2 labeled with green fluorescent protein (GFP), they found that upon stimulation with IL-12, IL-18, and IL-33, the number of IL-18Rα^+^ ILC1 cells increased, and 50% of these cells expressed ST2-GFP. Collectively, these data suggest that lung ILC1s are derived from ILC2s that are resident in the lung rather than from ILC1 proliferation.

In the same study, GFP^+^ ILC2s (GFP-labeled ILC2s) were transferred to *Rag2* ^−/−^/*Il2rg* ^−/−^ mice with mature lymphocyte deficiency, and then the mice were infected with influenza A virus. GFP^+^ ILC2s infiltrated into the lungs of host mice 7 d after infection. GATA3 expression in these cells was significantly down-regulated and accompanied by a striking up-regulation of both IL-18Rα and IL-12Rβ2 expression. Double IHC (immunohistochemistry) revealed that ILCs were localized to the influenza virus-infected airways. IHC combined with hybridization *in situ* revealed that *Il-12* and *Il-18* mRNAs produced by myeloid-derived cells were present near GFP^+^ ILC2s in the inflamed region. GATA3^high^ILCs were predominantly localized in uninfected tissue regions, whereas GATA3^low^ ILCs were enriched in virus-associated areas (Silver et al., 2016a).

**Figure 2:**
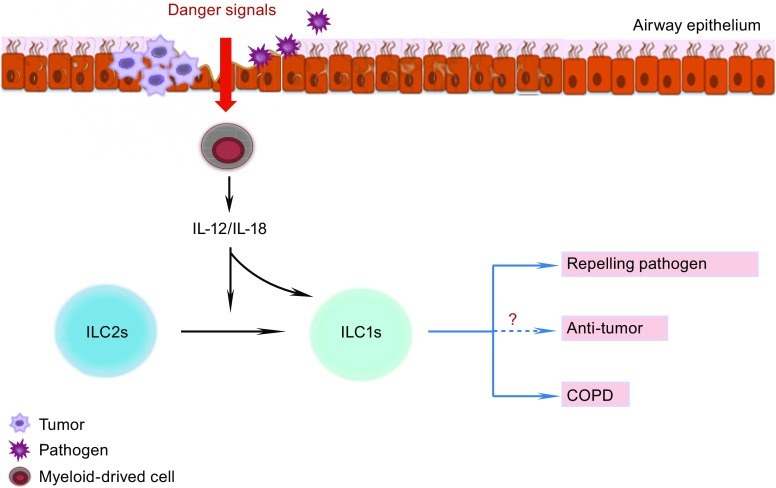
**ILC1 functions in the lung**. When pathogens, such as viruses or bacteria, or tumor cells invade the airway epithelium, the myeloid cells receive danger signals from the epithelium and produce IL-12 and IL-18. These pro-inflammatory cytokines down-regulate GATA3 expression of ILC2s and then drive the conversion of ILC2s into ILC1s. IL-12 and IL-18 also enhance the activation and expansion of ILC1s. After activation, ILC1s produce copious amounts of IFN-γ. IFN-γ plays potentially important roles in clearing both pathogens and tumors, and also in the development of chronic obstructive pulmonary disease (COPD). See text for details

In summary, these data demonstrate that during infection, ILC2s migrate to the inflamed regions, where the myeloid-derived pro-inflammatory cytokines IL-12 and IL-18 drive ILC2 conversion into ILC1s, enabling their participation in the anti-pathogen response (Fig. 2).

### ILC1s and chronic obstructive pulmonary disease (COPD)

COPD is widely regarded as a heterogeneous disease associated with increased numbers of alveolar macrophages, T lymphocytes (predominantly Tc1, Th1, and Th17 cells), B lymphocytes, and neutrophils (Barnes, 2009; Kearley et al., 2015). Recently, two groups almost simultaneously reported a relationship between ILC1s and COPD (Bal et al., 2016; Silver et al., 2016a). The percentage of ILC1s is much higher in patients with COPD than in healthy controls, and is accompanied by a lower occurrence of ILC2s, either in the lung or in the circulation (Bal et al., 2016; Silver et al., 2016a). According to the classification of the Global Initiative for Chronic Obstructive Lung Disease (GOLD), ILC1s occur more frequently in severe COPD (GOLD III–IV) than in milder COPD (GOLD I–II). A strong negative correlation exists between the occurrence of ILC1s in the blood and lung function, with a higher proportion of ILC1s associated with worse lung function. The numbers of circulating ILC1s are higher in patients with two or more exacerbations of COPD per year than in patients with one exacerbation per year (Bal et al., 2016; Silver et al., 2016a).

The development and exacerbation of COPD are associated with cigarette smoke and viral and bacterial infection. Silver et al. (2016a, b) reported that the occurrence of GATA3^+^ ILC2s declines promptly and that the fraction of T-bet^+^IL-18Rα^+^ ILC1s is increased in response to cigarette smoke or viral and bacterial infections in mouse models (Silver et al., 2016b). When ILC2s from human fetal lung are cultured with IL-2, IL-1β, and IL-12, CRTH2 and c-kit in ILC2s are down-regulated, and the cells produce IFN-γ but not IL-5. These results indicate that ILC2s have the potential to transform into ILC1s when exposed to a type 1 inflammatory environment, such as cigarette smoke or infection, and participate in the development of COPD (Bal et al., 2016) (Fig. 2).

### ILC1s and tumors

Recently, Dadi et al. (2016) discovered that unconventional type 1-like innate lymphoid cells and type 1 innate-like T cells play a role in tumor-elicited immune surveillance in murine cancer models (Dadi et al., 2016). This suggests that ILC1s may possess an anti-tumor function; however, no data on the function of ILC1s in lung tumors are available, and future studies should thus be performed to further elucidate this issue.

## Identification and characterization of ILC2s

In 2001, Fort et al. were the first to report that non-T/non-B T *cells in Rag2* ^−/−^mice could produce IL-5 and IL-13, leading to a type 2 response (Fort et al., 2001). Ten years later, these cells were observed by different researchers and were differently named as nuocytes, natural helper cells, or innate helper type 2 cells (Moro et al., 2010; Neill et al., 2010; Price et al., 2010). These three groups of cells share similar molecular surface markers and function, and finally they were collectively named as group 2 innate lymphoid cells. In addition to Id2 and IL-2Rγ, the development and function of ILC2s depend on GATA3, Notch, and RORα (Halim et al., 2012b; Mjosberg et al., 2012; Gentek et al., 2013). Growth factor independence-1 (Gfi1), B cell leukemia/lymphoma 11b (Bcl11b), lysine methyltransferase G9a, and ETS1 are also essential for the development of ILC2s (Spooner et al., 2013; Walker et al., 2015; Yu et al., 2015; Zook et al., 2016).

ILC2s can be activated by IL-25, IL-33, and thymic stromal lymphopoietin (TSLP), which are produced by epithelial cells and certain immune cells. Other ILC2 activators were later identified, namely, tumor necrosis factor (TNF)-family cytokine TL1A, prostaglandin D_2_, cysteinyl leukotriene receptor 1 (CysLT1), arginase 1, receptor for advanced glycation end-products, and surfactant protein D (Barnig et al., 2013; Doherty et al., 2013; Meylan et al., 2014; Yu et al., 2014; Tait Wojno et al., 2015; Taniguchi et al., 2015; Monticelli et al., 2016; Thawer et al., 2016). IL-1β has been considered to be an activator of ILC3s, but a recent study demonstrated that IL-1β may also activate ILC2s (Bal et al., 2016). These cells can also produce amphiregulin to enhance the recovery of the mucosa during viral infection (Monticelli et al., 2011). ILC2 inhibitors were recently identified and include prostaglandin I_2_, IFN-γ, IL-27, and lipoxin A4. Because these molecules inhibit ILC2 proliferation and cytokine production (McHedlidze et al., 2016; Moro et al., 2016; Zhou et al., 2016), they may be used to control ILC2-related diseases (See below). Activated ILC2s predominantly produce type 2 cytokines such as IL-5, IL-13, and IL-4, and also IL-9 (Moro, 2010; Mjosberg et al., 2011; Wilhelm et al., 2011; Kim et al., 2013).

In the mouse lung, ILC2s are defined as Lin^−^CD90^+^ICOS^+^CD25^+^ST2^+^CD127^+^. They also express CD44 and IL-17BR, and 20% of nuocytes (ILC2s) express c-kit (CD117) (Monticelli et al., 2011; Barlow et al., 2012; Bartemes et al., 2012). Based on their killer cell lectin-like receptor G1 (KLRG1) expression, mouse lung ILC2s can be divided into two groups: nILC2s, designated as Lin^−^ST2^+^KLRG1^int^ cells, and iILC2s, designated as Lin^−^ST2^−^KLRG1^hi^ (Huang et al., 2015). ILC2s are also found in the fetal and adult lung, and in the bronchoalveolar lavage fluid (BLF) in human. They are defined as Lin^−^CD127^+^CD161^+^CRTH2^+^ and also express ICOS, CD25 and ST2; they also partially express CD117 (Mjosberg et al., 2011; Monticelli et al., 2011). ILC2s in mouse and human have similar markers and function, although CRHT2 is specific for human since mouse ILC2s do not express this marker (Table 2).

## ILC2s in the lung

ILC2s were the first ILC group identified in lung (Monticelli et al., 2011). ILC2-related diseases in the lung involve pathogen infections (virus and helminth parasites), asthma, pulmonary fibrosis, and eosinophilic pleural effusion in primary spontaneous pneumothorax.

### ILC2s and viral infection

**Figure 3:**
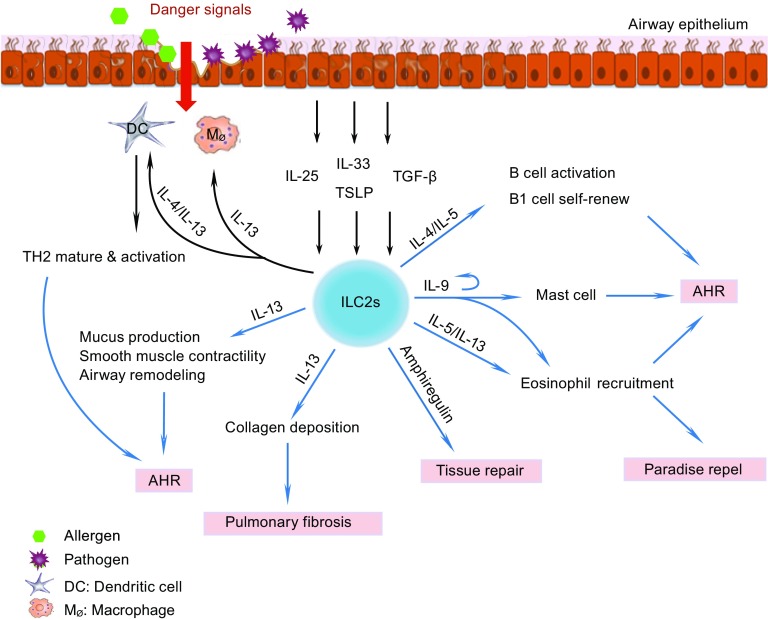
**ILC2 functions in the lung**. Following interaction with pathogens or allergens, the airway epithelium secretes IL-25, IL-33, TSLP, and TGF-β. Upon a molecular cue from the damaged epithelium, activated macrophages and DCs also release IL-33. All of these cytokines activate ILC2s. After activation, ILC2s proliferate and produce copious amounts of IL-4, IL-5, IL-9, IL-13, and amphiregulin. IL-4 stimulates B cells and also activates DCs and enhances Th2 cell maturation and activation. IL-5 and IL-13 stimulate the proliferation and recruitment of eosinophils, which are involved in parasite clearance and airway hyper-responsiveness (AHR). IL-5 also enhances B1 cell self-renewal and antibody production by B cells. ILC2s produce IL-9, and the autocrine effect of IL-9 stimulates secretion of effector cytokines by ILC2s. IL-13 induces smooth muscle contractility and airway remodeling that leads to AHR. Additionally, IL-9 plays an essential role in mast cell differentiation and also can induce mast cells to secrete IL-6; IL-9 can also promote both the proliferation of eosinophils and induce their migration into the lung. IL-13 can also induce collagen deposition and the development of pulmonary fibrosis. Furthermore, IL-13 enhances eosinophil recruitment and alternative macrophage activation. Finally, ILC2s also play an important role in airway remodeling by secreting amphiregulin. See text for details

During viral infection, ILC2s may exert a dual effect in the lung: on the one hand, ILC2s play a protective role in repelling the virus; on the other hand, if ILC2 function is not tightly controlled, these cells may induce airway hyperactivity (Fig. 3).

#### Maintenance of epithelial integrity

During the early phase of respiratory infection caused by influenza A virus subtype H_1_N_1,_ no significant differences were observed between wild-type mice and *Rag* ^−/−^ mice that lack adaptive immunity with respect to the decline in lung function and pathology of the lung. Because ILC2s accumulated in the lung, it was inferred that lung ILC2s play a key role in the regulation of lung innate immunity and tissue homeostasis (Monticelli et al., 2011). Depletion of ILCs led to substantial lung epithelial degeneration and necrosis, as a result of significantly impaired epithelial integrity. Adoptive transfer of ILCs to ILC-depleted mice effectively restored epithelial integrity (Monticelli et al., 2011). In the same study it was found that IL-33/IL-33R signaling was essential for the accumulation of ILC2s in H_1_N_1_ virus infected lungs whereas IL-13 and IL-22 appeared to be dispensable for tissue homeostasis (Monticelli et al., 2011). Data from genome-wide transcriptional profiling has suggested that ILC2s in the lung express wound healing-associated genes at higher levels. *In vivo*, lung ILC2s have the ability to produce high levels of amphiregulin, a molecule that regulates tissue remodeling and repair during acute epithelial injury and asthma (Dolinay et al., 2006; Enomoto et al., 2009; Fukumoto et al., 2010; Monticelli et al., 2011). In summary, lung ILC2s play an important role in maintaining the integrity of the respiratory epithelium and restoring lung function by producing amphiregulin during H_1_N_1_ infection.

#### Induction of lung inflammation and airway hyper-reactivity

Chang et al. (2011) observed that BALB/c mice infected with H_3_N_1_ virus rapidly develop AHR (airway hyper-responsiveness) that peaked on day 5 of infection. Because the sensitization of Th2 cells and enrollment of adaptive immunity during allergen-induced AHR required 7~14 d to develop (Hansen et al., 1999), based on the speed with which H_3_N_1_ induced AHR, it was hypothesized that this response was mediated by innate immune mechanisms. The authors provided evidence that indeed H_3_N_1_-induced AHR development did involve the innate immune pathway and did not require T, B, or NKT cells (Chang et al., 2011). Depletion of natural helper cells suppressed the AHR response, and furthermore only the transfer of IL-13-producing ILC2s, but no other IL-13-producing cells (such as mast cells or basophils), was sufficient for the development of H_3_N_1_-induced AHR. Furthermore, H_3_N_1_-induced AHR was tightly correlated with the presence of ILC2s, IL-33, and IL-13. IL-33 derived from alveolar macrophages likely plays an essential role in the activation of ILC2s (Chang et al., 2011). These data confirm that ILC2s are essential for the development of AHR during H_3_N_1_ infection.

During RSV infection, the number of IL-13-producing ILC2s triples compared with basal levels, and the IL-13 levels increase simultaneously. This increase contributes to airway hyper-reactivity and airway mucus accumulation in a TSLP-dependent manner (Stier et al., 2016). ILC2s have also been shown to produce IL-13 in order to recruit eosinophils and induce AHR through the IL-33/ST2 pathway during RSV infection (Liu et al., 2015b). Similarly, neonatal rhinovirus induces AHR and mucus metaplasia through IL-25 and ILC2s (Hong et al., 2014).

### ILC2s and helminth parasites

Upon infection with helminthic parasites, the host undergoes a strong type 2 response to clear the pathogens (Paul and Zhu, 2010; Maizels et al., 2012). During *Strongyloides venezuelensis* infection of the lung, IL-33 levels increase, activating ILC2s to release the effector cytokines IL-5 and IL-13 that, in turn, can recruit eosinophils to fight the infection. IL-33^−/−^ mice are unable to recruit eosinophils to the lungs or expel *S. venezuelensis* (Yasuda et al., 2012). During lung inflammation induced by *Nippostrongylus brasiliensis*, epithelial cells produce TSLP and IL-33, synergistically stimulating ILC2s to produce IL-5 and IL-13. Furthermore, IL-9, which is produced by ILC2s in an autocrine manner, stimulates IL-5 and IL-13 production by ILC2s. In IL-9 receptor-deficient mice infected with *N. brasiliensis*, ILC2s numbers are reduced and IL-5, IL-13, and amphiregulin levels are decreased, whereas the numbers of Th2 cells remain unchanged. As a result, helminth clearance is strongly impaired (Turner et al., 2013). The IL-9 signal plays an important role in the activation of ILC2s, especially in the early phase of helminth infection. This process also requires interferon regulatory factor 4 (IRF4) (Turner et al., 2013; Mohapatra et al., 2016).

During *N. brasiliensis* infection, the ILC2 subgroup iILC2 can convert into nILC2. *N. brasiliensis*-infected *Il17rb* ^−/−^ mice lack iILC2 cells and, interestingly, nILC2 cell numbers are significantly decreased in these mouse models. This suggests that iILC2s contribute to the nILC2 population during *N. brasiliensis* infection. Researchers simultaneously transferred CD45.1^+^ nILC2s and CD45.2^+^ iILC2s into *Rag2* ^−/−^ *Il2rg* ^−/−^ mice infected with *N. brasiliensis*, and 14 d later found that all iILC2 cells had developed into nILC2-like cells. iILC2s may thus comprise a transient progenitor population of cells that can transform into nILC2-like cells and be involved in the clearance of *N. brasiliensis* (Huang et al., 2015) (Fig. 1).

Interestingly, another natural mouse parasite, *Heligmosomoides polygyrus*, that causes parasitic infections suppresses inflammatory responses in models of asthma, food allergy, diabetes, and colitis (McSorley and Maizels, 2012). *H. polygyrus* excretory/secretory (HES) products can suppress both Treg (Grainger et al., 2010) and dendritic cells (Massacand et al., 2009). McSorley et al. found that HES products inhibit the allergic reaction in the ovalbumin (OVA)-induced mouse asthma model by suppressing the release of IL-33 and inhibiting the activation of ILC2s (McSorley et al., 2014; McSorley et al., 2015).

### ILC2s and asthma

Asthma is a heterogeneous disease that occurs worldwide and whose pathology involves chronic airway inflammation. The type 2 response is regarded as a central mechanism for allergic asthma (Deckers et al., 2013; Licona-Limon et al., 2013). Conventionally, Th2 cells have been regarded as the main source of type 2 cytokines, but this notion has now been challenged by the emergence of ILC2 cells.

In mouse asthma models that use ovalbumin (Kim et al., 2012), house dust mite (HDM) (Wilhelm et al., 2011; Halim et al., 2012a; Klein Wolterink et al., 2012), papain (Halim et al., 2012a), and fungal allergens, ILC2s uniformly increase in number and are the major source of IL-5 or/and IL-13, especially in the early phases of the disease. In mouse models that lack adaptive immune cells, i.e., T and B cells, allergens can also induce significant AHR, a high level of type 2 cytokines, and increased numbers of ILC2s in the lung (Bartemes et al., 2012; Halim et al., 2012a). In these allergen-induced asthma models, the pulmonary epithelium, macrophages, and DCs release IL-33; in addition, the pulmonary epithelium also produces IL-25, TSLP, and transforming growth factor-β (TGF-β). All of these cytokines can activate ILC2s (Halim et al., 2012a; Kim et al., 2012; Iijima et al., 2014; Denney et al., 2015). *In vitro*, IL-25 and IL-33 enhance the proliferation of ILC2s and stimulate them to produce IL-4, IL-5, IL-13, and/or IL-9 (Wilhelm et al., 2011; Barlow et al., 2012; Bartemes et al., 2012; Halim et al., 2012a; Kim et al., 2012; Klein Wolterink et al., 2012). This is important because, IL-4 stimulates B cells to produce IgE, IL-5 recruits and activates eosinophils and also enhances B cell antibody production and B1 cell self-renewal, and IL-13 enhances smooth muscle contraction, epithelial mucous production, airway remodeling, and eosinophil recruitment. IL-9 plays an essential role in mast cell differentiation and can also induce mast cells to secrete IL-6; IL-9 promotes both the proliferation of eosinophils and their migration into the lung (Renauld, 2001). Otherwise, the IL-9 production of ILC2s depends on IL-2 secreted by adaptive immune cells. IL-9 also stimulates ILC2s to produce IL-13 and IL-5 (Wilhelm et al., 2011). All of these cytokines play an important role in the development of AHR (Fig. 3).

During AHR development, ILC2s crosstalk with DCs, CD4^+^ Th2 cells, B cells, and Th9 cells, thereby potentiating the pathology. ILC2s produce IL-13, which directly activates DCs to express CCL17, enhancing CD4^+^ Th2 cell activation; ILC2s also activate Th2 cells directly by producing IL-4 and OX40L. ILC2s and CD4^+^ Th2 cells thereby exert a synergistic effect on the development of AHR (Drake et al., 2014; Gold et al., 2014; Halim et al., 2014; Halim et al., 2015; Liu et al., 2015a). Mouse lung ILC2s enhance the proliferation of B1- and B2-type B cells and stimulate their production of IgM, IgG1, IgA, and IgE *in vitro*. Specifically, ILC2-derived IL-5 is critically involved in increased IgM production (Drake et al., 2016). Polarized ILC2s and Th9 cells also stimulate each other in mouse models of asthma in the lung (Ying et al., 2016).

Researchers have recently observed that similar to Th2 cells, ILC2s gain memory-like properties upon allergen challenge. ILC2s stimulated by inhalation of either IL-33 or papain persist for a long time; even after resolution of the inflammatory response; in fact, some ILC2s persist for more than 4 weeks. Furthermore, ‘allergen-experienced’ ILC2s responded better to unrelated allergen than naïve ILC2s, mediating a more severe allergic inflammation (Martinez-Gonzalez et al., 2016). Allergen-experienced ILC2s are also more responsive and produce higher amounts of the same cytokines than unexperienced ILC2s. Compared with memory lymphocytes, ILC2s are activated by cytokines, while memory lymphocytes are activated by specific antigens, thus the memory-like ILC2s are antigen non-specific. This means that memory-like ILC2s can be activated by unrelated allergens but produce a stronger response than naïve ILC2s. Memory-like ILC2s can also enhance Th2 cell-mediated adaptive type 2 lung inflammation. These two important characteristics may explain the phenomena that some asthma patients react against multiple allergens, while some do not (Martinez-Gonzalez et al., 2016).

In comparison with mouse, far fewer studies describing the role of ILC2s in human asthma have been published. The occurrence of ILC2s is more frequent in the blood of subjects with allergic asthma than in healthy individuals and allergic donors. When stimulated with IL-25 or IL-33, peripheral blood mononuclear cells from patients with allergic asthma produced significantly greater amounts of IL-5 and IL-13 than those from patients with allergic rhinitis and those from healthy donors (Bartemes et al., 2014; Jia et al., 2016). Recently, ILC2s were found in the bronchoalvoelar lavage (BAL) and sputum of patients with asthma, and the proportion of IL-5^+^IL-13^+^ ILC2s in the sputum of patients with severe asthma was higher than that in corresponding samples from patients with mild asthma (Smith et al., 2016). ILC2 levels are also increased in the sputum from severe asthmatic children (Nagakumar et al., 2016). Other researchers have also compared the relationship between ILC2s and asthma control status, and the results indicate a positive correlation between IL-13-producing ILC2s and asthma control status (Jia et al., 2016).

Based on the above studies of examining ILC2s in human asthma, the number of ILC2s is increased in asthmatic patients’ blood, these ILC2s are activated, and the number of IL-13-producing ILC2s negatively correlates with asthma control status. These results might suggest that level of ILC2s in the blood maybe partially indicate asthma status. However, ILCs are generally tissue-resident cells and the characteristics of ILCs in tissue are very different from ILCs in the circulation since tissue-resident ILCs are affected by the tissue microenvironment, which can cause changes of biomarker profile as well as function. Therefore, the blood ILC profile and function does not absolutely represent ILC2s resident in the lungs of asthmatics; blood ILC2s therefore only partially indicates the status of the disease. In the future, it will be important to study the function of human tissue-resident ILC2s, for example ILC2s from BAL and sputum, and even from lung tissue itself.

### ILC2s and pulmonary fibrosis

Pulmonary fibrosis is a heterogeneous disease that is prevalent worldwide, and that is typically regarded as a chronic progressive disease, with high morbidity and mortality (Hutchinson et al., 2015). The key pathogenic mechanism involves extracellular matrix deposition in the lung. The pro-inflammatory cytokines TGF-β, IL-13, IL-1β, and IL-17A all play an important role in the fibrotic process (Kolb et al., 2001; Wynn, 2011).

In the mouse model of pulmonary fibrosis that uses injection of *S. mansoni* eggs, IL-25 has been shown to be the key cytokine in the development of fibrosis. IL-25 induces a dramatic increase in both IL-13 and TGF-β in the lungs. In humans, increased levels of IL-25 and ILC2s are found in the BAL and lung tissue of patients with idiopathic pulmonary fibrosis (Hams et al., 2014). Compared with wild-type mice, pulmonary collagen deposition is impaired in ILC2-deficient mice after *S. mansoni* egg injection (Hams et al., 2014). Furthermore, ILC2s induce pulmonary collagen deposition in an IL-13-dependent manner (Hams et al., 2014). Li et al. (2014) have suggested that the IL-33-ST2 axis is essential for the initiation and progression of pulmonary fibrosis. In this model IL-33 activates M2 macrophages to produce IL-13 and TGF-β1, and then further induces the expansion of ILC2s to produce IL-13, ultimately resulting in the development of pulmonary fibrosis (Li et al., 2014).

Dermal and circulating ILC2 counts correlate closely with the occurrence of pulmonary fibrosis in systemic sclerosis patients. This implies that ILC2s may aggravate the pulmonary fibrosis in these patients (Wohlfahrt et al., 2016) (Fig. 3).

### ILC2s and eosinophilic pleural effusion (EPE) in primary spontaneous pneumothorax (PSP)

EPE is defined as > 10% eosinophilia in the pleural fluid and is frequently associated with the presence of blood and/or air in the pleural space (Kalomenidis and Light, 2003). PSP is a common complication, with a high rate of recurrence in EPE (Kalomenidis and Light, 2003).

The levels of IL-4, IL-5, IL-13, and Eotaxin-3, as well as TSLP and IL-33, have been shown to be increased in the pleural fluid of PSP patients. These cytokines are type 2 immune response-related, and it therefore appears that Th2 cells and ILC2s might play an essential role in this pathology; however, CD4^+^ and CD8^+^ T cells are known not to be involved in the pathogenesis of PSP (Kwon et al., 2013). IL-33 directly stimulates ILC2s to produce increased amounts of IL-5, which then recruits eosinophils into the pleural space, resulting in EPE; Th2 cells are not involved in this process. This indicates that the type 2 immune response is associated with the development of EPE in PSP through an ILC2-dependent but Th2-independent manner (Kwon et al., 2013).

## Identification and characterization of ILC3s

ILC3s can be classified into two main groups according to the host developmental stage when they mature: fetal LTi cells and post-natal (adult) ILC3s. Fetal LTi cells, the prototypical ILC3s, were originally reported two decades ago. In mice, LTi cells are identified as CD127^+^CD3^−^CD4^+^. Their development is RORγt-dependent, they originate in the fetal liver, and they are found in fetal lymphoid nodes and the intestine (Kelly and Scollay, 1992; Mebius et al., 1997; Yoshida et al., 1999; Eberl et al., 2004; Finke, 2005). In humans, fetal LTi cells were first found in 2009 and recognized as Lin^−^RORγt^+^CD127^+^CD4^−^; they play a role similar to that of mouse LTi cells (Cupedo et al., 2009).

In mice, adult ILC3s are defined as CD45^+^Lin^−^Thy1^+^RORγt^+^, and they partially express CCR6 and NKp46 (Sanos et al., 2009; Takatori et al., 2009; Vonarbourg et al., 2010b; Song et al., 2015). RORγt, AHR, GATA3, and T-bet are required for their development (Luci et al., 2009; Klose et al., 2013; Hughes et al., 2014; Serafini et al., 2014). In humans, adult ILC3s are defined as Lin^−^CD127^+^CRTH2^−^CD117^+^; they also heterogeneously express NKp44, NKp46, CD56, and NKp30 (Crellin et al., 2010; Hoorweg et al., 2012; Glatzer et al., 2013). Upon stimulation with IL-1β and IL-23, ILC3s express IL-17, IL-22, and granulocyte-macrophage colony-stimulating factor (GM-CSF) (Takatori et al., 2009; Crellin et al., 2010; Hoorweg et al., 2012; Song et al., 2015).

Adult ILC3s are the most heterogeneous ILCs. Depending on the level of the expressed NK receptor, e.g., NKp44, NKp46, or NKp30, ILC3s can be divided into NCR^−^ILC3s and NCR^+^ILC3s. ILC3s can also be divided according to the level of expression of CCR6 into CCR6^−^ ILC3s and CCR6^+^ ILC3s. Recently, it was shown that NCR engagement enables ILC3s to play a pro-inflammatory role (Glatzer et al., 2013); based on this an increasing numbers of researchers use the NCR status to classify the function of adult ILC3s. Similar markers and functions are found in both mouse and human ILC3s with the exception that mice do not express NKp44 (Killig et al., 2014).

In the mouse lung, ILC3s were initially reported by Centre et al. (2015), who found that 30% of ILCs were ILC3s, defined as Lin^−^CD90^+^CD127^+^RORγt^+^; almost 70% of ILC3s also co-express CCR6. IL-1β and IL-23 have been shown to activate these cells. ILC3s were also identified as a major source of IL-22 produced in response to IL-23 stimulation (Van Maele et al., 2014). In the human lung, ILC3s are identified as Lin^−^CD127^+^CRTH2^−^CD117^+^, and are NCR^−^ or NCR^+^. NCR^+^ILC3s produce IL-22, TNF-α, IL-8, IL-2, and GM-CSF upon stimulation (Carrega et al., 2015; De Grove et al., 2016) (Table 2).

## ILC3s in the lung

### ILC3s and infection

ILC3s play an essential role in the maintenance of mucosal barrier function because they produce effector cytokines, especially IL-22 and IL-17. IL-22 and IL-17 activate epidermal cells to produce antimicrobial molecules and protect the host from extracellular bacteria and fungi (Liang et al., 2006). IL-22 can also enhance the epithelial production of mucus-associated molecules (McAleer and Kolls, 2014). A protective role of ILC3s against infections in the digestive system has also been reported. During an intestinal infection with *Citrobacter rodentium*, the number of IL-22-producing ILC3s increased in the intestinal lamina propria (Cella et al., 2009; Sanos et al., 2009). Gladiator et al. found that ILC3s can also produce IL-17 that helps eliminate pathogens during fungal infection in mice, especially in the early phase of infection. (Gladiator et al., 2013). In 2014, Van Maele et al. reported the function of ILC3s in the lung. During *Streptococcus pneumoniae* infection, ILC3s rapidly accumulate in the lung tissue to produce IL-22 in a DC- and MyD88-dependent manner (Van Maele et al., 2014). Boosting lung ILC3 numbers might therefore represent an interesting strategy for fighting respiratory bacterial infections.

One study suggested that Toll-like receptor 5 (TLR5) signaling could activate ILCs (CD3^−^CD127^+^) and enhance the production of IL-17 and IL-22, which are crucial for anti-pathogen defenses in the mucous membrane of the intestine and the lung (Van Maele et al., 2010). This raises the interesting notion that TLR5 may activate ILC3s to reject the pathogen in the lung. Later, Van Maele et al. found that during *S. pneumoniae* infection, the TLR5 agonist flagellin accelerates and over-stimulates lung ILC3s to produce more IL-22 (Van Maele et al., 2014).

**Figure 4:**
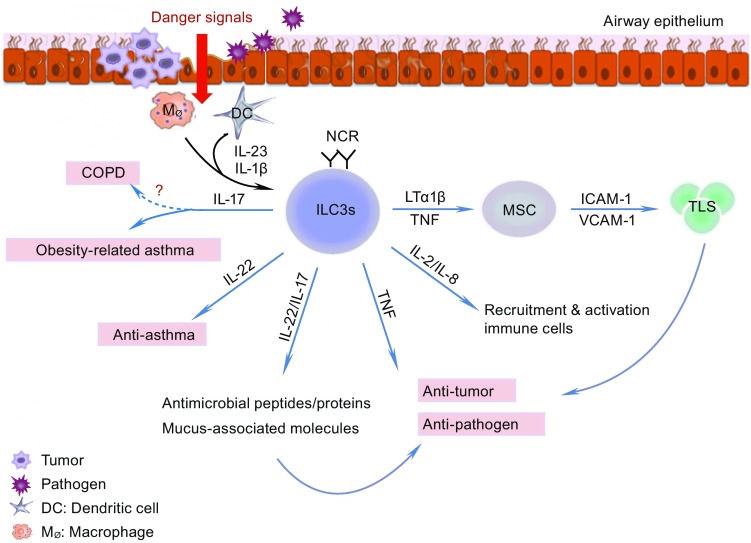
**ILC3 functions in the lung**. After receiving the danger signals from the airway epithelium upon pathogen and tumor cell invasion, DCs and macrophages release IL-1β and IL-23, which activate ILC3s. Activated ILC3s release IL-17, IL-22, IL-8, IL-2, TNF, and LTα1β2. IL-17 and IL-22 play a dual role: on the one hand, they stimulate the epithelium to produce antimicrobial peptides and proteins, and mucus-associated molecules to clear the pathogen and tumor cells to maintain homeostasis of the mucosa; on the other hand, they may aggravate the development of obesity-related asthma and COPD. IL-22-producing-ILC3s may have anti-asthma effects, whereas IL-17-producing ILC3s may participate in the pathology of asthma, especially in obesity-related asthma. IL-2 and IL-8 recruit neutrophils to the lung. LTα1β2 stimulates mesenchymal stem cells (MSCs) to express ICAM-1 and VCAM-1. These molecules participate in the formation of a tertiary lymphoid organ in the tumor. See text for details

*Klebsiella pneumoniae* is a gram-negative bacterium that is highly resistant to antibiotics and is a common pathogen in pneumonia (Doorduijn et al., 2016). In 2014, Xu et al. found that IL-22-producing NK cells are required for optimal host defense in mouse models of *K.* *pneumoniae* infection (Xu et al., 2014). Recently, another group reported that following infection with *K.* *pneumoniae* in mice, inflammatory monocytes are immediately recruited to the lungs, where they produce TNF, which then increases the number of IL-17-producing ILC3s. IL-17A-dependent clearance of *K.* *pneumoniae* is impaired in monocyte- or TNF-depleted mouse models, whereas IL-17-producing ILC3s enhance monocyte-mediated bacterial clearance. These results indicate that ILC3s and monocytes participate in a positive feedback cycle that promotes the clearance of highly antibiotic-resistant bacterial pathogens from the lung (Xiong et al., 2016) (Fig. 4).

iILC2-derived ILC3-like cells also play a role in *Candida albicans* infections. iILC2s express an intermediate amount of RORγt, i.e., one that is significantly different from nILC2s but lower than that in ILC3 cells. A small proportion of freshly isolated iILC2s produces IL-17 upon stimulation with PMA and ionomycin (Huang et al., 2015). When cultured with TGF-β and IL-6, iILC2s become ILC3-like, produce IL-17, and lose the ability to produce IL-13. During *C. albicans* infection, iILC2 cells aid in the clearance of this pathogen. In the lungs of mice infected with *C. albicans*, transferred iILC2 cells become ILC3-like cells after 5 d; these ILC3-like cells produce IL-17 but not IL-13 (Huang et al., 2015). Thus, iILC2 cells can transform into ILC3-like cells *in vitro* and *in vivo*, and gain the ability to protect the host against *C. albicans* (Huang et al., 2015) (Fig. 1).

### ILC3s and asthma

In addition to ILC2s, ILC3s are also involved in asthma. In an OVA-induced asthma murine model, Taube et al. found that IL-22 expression increased, and the IL-22 was mainly produced by innate lymphoid cells in the lungs, rather than by TH cells. OVA challenged IL-22-deficient mice suffered from much higher AHR. In contrast mice treated with IL-22 before OVA challenge displayed significantly reduced allergic airway inflammation. Based on these data, IL-22-producing ILC3s may participate in reducing allergic asthma pathology (Taube et al., 2011).

Obesity is a risk factor associated with asthma, and obese asthma patients respond poorly to typical anti-asthma medications, including corticosteroids (Sutherland et al., 2009); therefore, a distinct immune mechanism must be at play in obese asthmatics. Mice fed a high-fat diet become obese and exhibit AHR through an IL-17A and NKRP3-dependent pathway, and this AHR also occurs in obese *Rag1* ^−/−^ mice (Kim et al., 2014). In this model, the number of CCR6^+^ IL-17A-producing ILC3s is elevated in the lung and macrophage-derived IL-1 directly causes AHR by stimulating this IL-17-producing ILC3 population (Kim et al., 2014).

Everaere et al. found a similar result where, compared with lean mice, the number of ILCs was increased in the lung of obese mice, and this effect was accompanied by eosinophil infiltration. Following an HDM challenge, the counts of ILC2s and ILC3s in the lung further increased, as did IL-33 and IL-1β levels, whereas ILC markers in visceral adipose tissue decreased. In an obese mouse model with ILC depletion, the HDM-induced inflammatory profile of the airway was profoundly decreased, including reduced Th2 and Th17 infiltration (Everaere et al., 2016) (Fig. 4).

In humans, IL-17-producing ILCs are found in BAL fluid samples from asthma patients; their levels are increased in severe asthma patients compared with those in patients with mild asthma or control donors (Kim et al., 2014).

These results indicate that IL-22-producing-ILC3s may have an anti-asthma effect, whereas IL-17-producing ILC3s participate in the pathology of asthma, possibly providing a link between obesity and asthma. Further studies should be performed to help illuminate these potential connections.

### ILC3s and COPD

IL-17A plays an essential role in the development of COPD. Since ILC3s have the ability to produce IL-17A it is therefore possible that ILC3s are linked with COPD. De Grove et al. (2016) found that in patients with COPD, the population of NCR^−^ILC3s comprises the largest subset of ILCs (De Grove et al., 2016). Bal et al. (2016) also found that NKp44^−^ILC3 levels were increased, whereas the numbers of ILC2s and NKp44^+^ ILC3s were significantly diminished in lung tissue from patients with severe COPD (Bal et al., 2016). This supports our hypothesis that IL-17-producing ILC3s may play a role in COPD; however, the authors suggested that the accumulation of NCR^−^ILC3s in COPD could be associated with the protective immunity of the host in response to bacterial respiratory tract infections that occur frequently in patients with COPD. The involvement of ILC3s in COPD therefore requires further study (Fig. 4).

### ILC3s and tumors

A dual effect of ILC3s on tumor immunity was suggested for intestinal tumors. On the one hand, IL-22 produced by ILC3s maintains mucosal integrity and clearance of pathogens and transformed cells. On the other hand, IL-22 activates the STAT3 cascade to enhance tumor generation (Kirchberger et al., 2013). Carrega et al. (2015) observed that NCR^+^ILC3s were enriched in non-small cell lung cancer (NSCLC) and that the proportion of NCR^+^ILC3s was positively associated with tumor stage. NCR^+^ILC3s were present in significantly higher amounts in stage I/II NSCLC tumors than in more tumors from more advanced stages. When stimulated, NCR^+^ILC3s, which had been freshly isolated from NSCLC tissues, produced IL-22, TNF-α, IL-8, and IL-2, but did not secrete IL-17. IL-22 maintains the integrity of epithelial cells (Dudakov et al., 2015); TNF-α is a pro-inflammatory cytokine that exerts anti-tumor and anti-pathogen effects, and IL-8 and IL-2 enhance leukocyte recruitment and proliferation (Waugh and Wilson, 2008; Boyman and Sprent, 2012). Based on the characteristics of the cytokines produced by NCR^+^ILC3s, it was concluded that these cells might play a role in anti-tumor defenses (Carrega et al., 2015).

As stated above, LTi is the prototype for ILC3s. LTi cells produce lymphotoxin α, lymphotoxin β, and TNF-α. They also stimulate stromal cells to produce vascular cell adhesion molecule 1 (VCAM-1) and intercellular adhesion molecule 1 (ICAM-1), which recruit the immune cells and form the fetal lymphoid node and Peyer’s patches (Kelly and Scollay, 1992; Mebius et al., 1997; Yoshida et al., 1999; Eberl et al., 2004; Finke, 2005; Cupedo et al., 2009). Lung cancer-derived NCR^+^ILC3s express more lymphotoxin mRNA than their tonsil counterparts. Furthermore, lung cancer-derived NCR^+^ILC3s elicit significant up-regulation of ICAM-1 and VCAM-1 in mesenchymal stem cells in a lymphotoxin α, lymphotoxin β, and TNF-α dependent manner. Additionally, NCR^+^ILC3s preferentially reside at the edge of lymphoid structures associated with NSCLC, and the percentage of NCR^+^ILC3s is correlated with the density of tertiary lymphoid structures in the tumor region. These observations suggest that NCR^+^ILC3s might play a role in the formation and maintenance of these structures as well as lymphoid aggregates in tumor tissue (Carrega et al., 2015). Otherwise, NCR^−^ILC3s can gain pro-inflammatory properties by engaging NKp44, as the number of NCR^+^ILC3s is increased in the lung tumor region (Fig. 4). Whether the conversion of NCR^−^ILC3 to NCR^+^ILC3 cells contributes to this increased number and how the tumor microenvironment influences this conversion are questions that should be answered in future studies. Additionally, the question of whether IL-22-producing ILC3s enhance or inhibit tumors in the lung remains unresolved and should be studied further.

## Conclusions

ILCs are attracting increasing attention on account of their distinct tissue-resident properties. Although it is known that ILCs are involved in pulmonary infection, asthma, COPD, fibrosis, and tumors in the lung, in-depth studies are still only in their infancy. The mechanisms of ILC activation, proliferation, and regulation in the lung are not clear; how exactly the pathological environment affects ILC function and how ILCs respond to the environment also remain unknown. Elucidation of these mechanisms is therefore an urgent matter, especially in pulmonary diseases. Answers to these questions will hopefully provide new clues for the treatment of these serious human diseases.
