# Erratum to: Guards at the gate: physiological and pathological roles of tissue-resident innate lymphoid cells in the lung

**DOI:** 10.1007/s13238-017-0399-1

**Published:** 2017-04-06

**Authors:** Hang Cheng, Chengyan Jin, Jing Wu, Shan Zhu, Yong-Jun Liu, Jingtao Chen

**Affiliations:** 1 Institute of Translational Medicine, The First Hospital, Jilin University, Changchun, 130061 China; 2 Department of Pediatrics, The First Hospital, Jilin University, Changchun, 130021 China; 3 Department of Thoracic Surgery, The Second Hospital, Jilin University, Changchun, 130041 China; 4 Sanofi Research and Development, Cambridge, MA 02139 USA

## Erratum to: Protein Cell DOI 10.1007/s13238-017-0379-5

In the original publication of this article on the 4th paragraph under the section “Identification and characterization of ILC3s” the line starting with, “In the mouse lung, ILC3s were initially reported by Centre et al. (2015)” is incorrectly published. The correct line is provided in this erratum as, “In the mouse lung, ILC3s were initially reported by Van Maele et al. (2014)”.

